# Schwannome du nerf sciatique: à propos d´un cas

**DOI:** 10.11604/pamj.2020.37.233.22745

**Published:** 2020-11-13

**Authors:** Aniss Chagou, Hamza Benameur, Jalal Hassoun, Abdeloihab Jaafar

**Affiliations:** 1 Mohammed VI University of Health Sciences (UM6SS), Casablanca, Morocco

**Keywords:** Schwannomme, nerf sciatique, sciatalgie, excision, *case report*, Schwannoma, sciatic nerve, sciatalgia, excision, case report

## Abstract

Les schwannomes du nerf sciatique sont des tumeurs rares. Elles se manifestent essentiellement par des névralgies sciatiques plutôt que par des déficits sensitivo-moteurs. Nous rapportons le cas d´une patiente de 30 ans présentant une douleur de la fesse droite depuis 1 an. La palpation a révélé un signe de Tinel positif. Une imagerie par résonnance magnétique (IRM) a été réalisée qui a objectivé une masse grossièrement arrondie, régulière de 2 cm de diamètre en contact intime avec le nerf grand sciatique. Une exérèse complète de la tumeur a été réalisée. Cette exérèse a permis la disparition de la douleur et l´examen anatomopathologique a conclu à un schwannome plexiforme.

## Abstract (en)

Schwannomas of the sciatic nerve are rare tumors. They are mainly manifested by sciatic neuralgia rather than by sensory-motor deficits. We report the case of a 30-year-old female patient with right buttock pain for 1 year. Palpation revealed a positive Tinel sign. A magnetic resonance imaging (MRI) was performed which objectified a roughly rounded, regular mass of 2 cm in diameter in intimate contact with the large sciatic nerve. A complete excision of the tumor was carried out. This excision allowed the pain to disappear and the pathology examination concluded with a plexiform schwannoma.

## Introduction

Les tumeurs des nerfs périphériques sont des tumeurs rares avec une incidence de 1/100000. La grande majorité de ces tumeurs sont des schwannomes. Il s´agit de tumeurs développées au dépend des cellules de Schwann. Les schwannomes siègent rarement au niveau du membre inférieur, l´atteinte du nerf sciatique est encore plus rare. Cette rareté conduit à une errance diagnostique.

## Patient et observation

**Figure 1:**
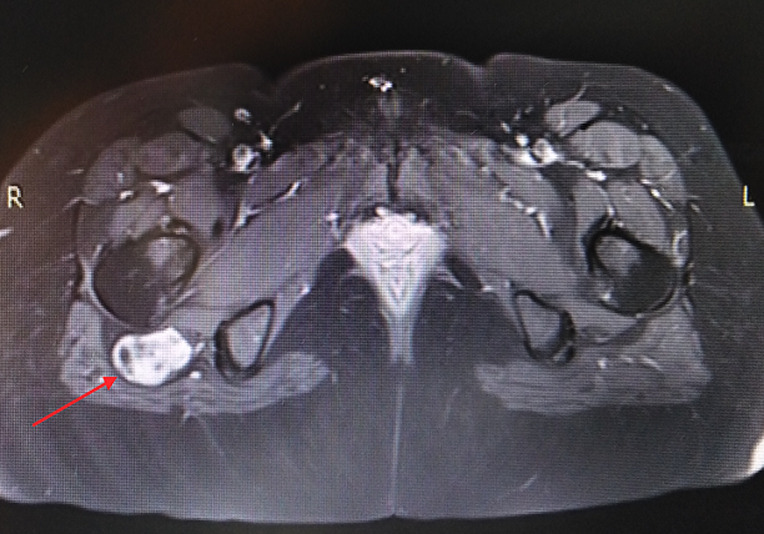
coupe axiale d'une IRM séquence T1, tumeur circonscrite prenant le contraste

**Figure 2:**
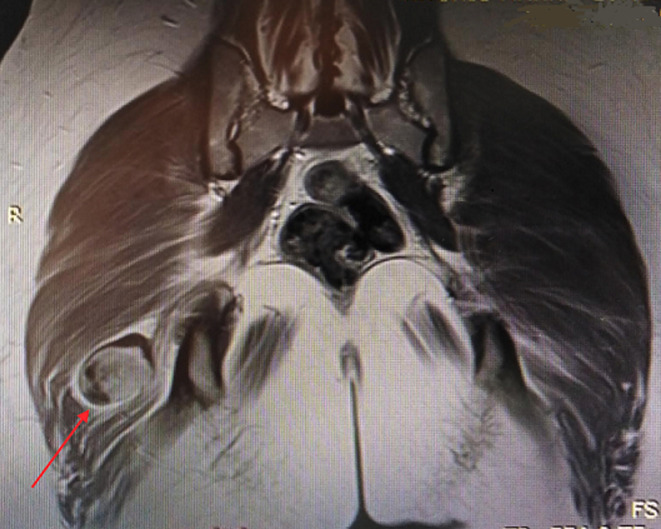
coupe coronale d'une IRM séquence T2 montrant la tumeur

**Figure 3:**
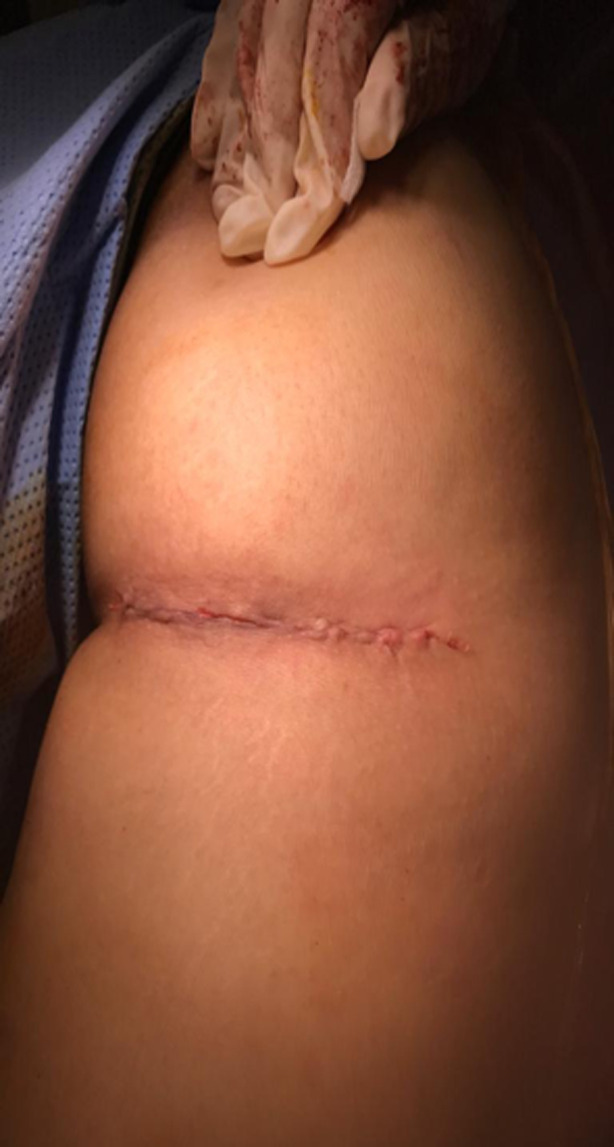
image de la cicatrice post-opératoire

**Figure 4:**
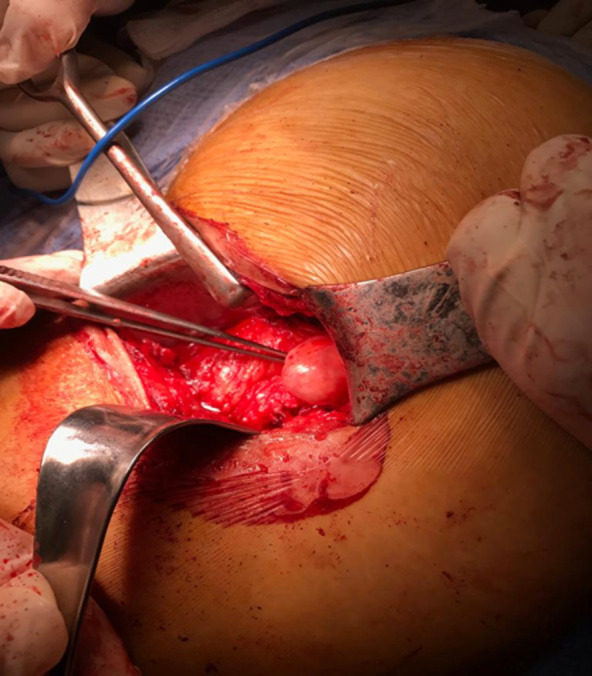
image peropératoire de la tumeur

Nous rapportons le cas d´une patiente âgée de 30 ans se plaignant depuis 1 an de douleurs de la fesse droite. Ces douleurs s´exacerbent en décubitus et en position assise. Elles sont majorées la nuit avec une sensation d´engourdissement. La palpation retrouvait un signe de Tinel au niveau de la fesse droite. L´échographie retrouvait en regard du signe de Tinnel une formation sur le trajet du nerf sciatique. Une IRM a été réalisée qui a objectivé une masse grossièrement arrondie, régulière de 2 cm de diamètre en contact intime avec le nerf grand sciatique (Figure 1, Figure 2). La résection de la tumeur a été réalisée en décubitus ventral, genoux en flexion afin de relâcher le nerf sciatique. Une incision transversale au niveau du pli fessier a été réalisée (Figure 3), les fibres du muscles grand fessier ont été écartés. Une exérèse complète de la tumeur a été pratiquée. Cette dernière était blanchâtre, encapsulée, au contact avec le nerf sciatique (Figure 4), Elle était également excentrique et facilement extirpable. Les suites étaient simples sans signes de déficit sensitivomoteur. Les douleurs rapportées initialement par le patient ont disparu. L´examen anatomopathogique a conclu à un schwannome bénin. A un recul de 18 mois, nous n´avons pas noté de récidive tumorale.

## Discussion

Les tumeurs des nerfs périphériques sont des tumeurs rares qui proviennent de la gaine des nerfs. On en distingue les schwannomes, les neurofibromes et les tumeurs malignes des nerfs (ou MPNST pour malignant peripheral nerve sheath tumors). Les schwannomes sont les tumeurs des nerfs périphériques les plus fréquentes (70%) [1]. Ils atteignent habituellement les grands troncs nerveux, en particulier, du membre supérieur. Au niveau du membre inférieur, le nerf le plus souvent atteint est le tibial postérieur [2]. Le nerf sciatique est rarement atteint, il représente moins de 1 % de tous les cas, Tout le trajet du nerf peut être le siège de cette tumeur [3]. Les schwannomes surviennent habituellement entre 20 et 50 ans [1]. La transformation maligne est rare. Ce sont des tumeurs encapsulées, bien définies, excentrées du trajet du nerf. Elles refoulent les fascicules nerveux, ce qui explique la pauvreté des symptômes neurologiques déficitaires malgré des lésions parfois volumineuses. Elles surviennent habituellement entre 20 et 50 ans, avec un sex-ratio proche de 1 [1,4]. Leur transformation maligne est rare [4].

Du fait d´une croissance lente, la durée des symptômes avant le diagnostic est souvent longue, pouvant atteindre plus de dix ans. Les signes cliniques les plus fréquemment observés sont des douleurs radiculaires et distales parfois très éloignées du site lésionnel (85 %), une masse palpable (43 à 96%) avec un signe de Tinel, des paresthésies (25 %) et plus rarement un déficit moteur [1-4]. A l´échographie, le schwannome apparaît comme une masse hypoéchogène, excentrée par rapport au nerf qui conserve sa structure fibrillaire conservée. Il présente en coupe transversale en forme de «cible ». Le neurofibrome représente le diagnostic différentiel. Contrairement au schwannome, il apparaît comme une masse solide bien centrée par rapport au nerf porteur dont la structure fibrillaire disparaît complètement [5,6].

L´IRM reste le gold standard en matière de diagnostic des schwannomes. Le schwannome se présente comme une masse excentrée sur le trajet du nerf ou de la racine, apparaissant en isosignal T1 et en hypersignal T2 [7]. L´IRM fait la différence entre un schwannome et un neurofibrome. Cette distinction est très importante, l´aspect envahissant du neurofibrome ne permet pas une dissection de la tumeur d´où le risque important de séquelles et de récidive. La chirurgie reste le traitement de choix pour le traitement des schwannomes. Le caractère limité de la tumeur permet dans la majorité des cas une exérèse complète. La lésion excentrée par rapport aux fascicules nerveux permet de disséquer ces derniers réduisant ainsi les séquelles postopératoires [1,3,5,8,9].

## Conclusion

Le schwannome du nerf sciatique est une tumeur rare, responsable de névralgies sciatique, il doit toujours être évoqué à chaque fois qu´aucune origine radiculaire n´a été trouvée. L´échographie et l´IRM permettent de différencier le schwannome d´un neurofibrome. Le caractère excentré de la tumeur permet en général une extirpation sans séquelles.
